# Immune Response to MVA-BN Vaccination for Mpox: Current Evidence and Future Directions

**DOI:** 10.3390/vaccines13090930

**Published:** 2025-08-30

**Authors:** Joanne Byrne, Patrick D. M. C. Katoto, Bruce Kirenga, Wilber Sabiiti, Andrew Obuku, Virginie Gautier, Patrick W. G. Mallon, Eoin R. Feeney

**Affiliations:** 1 Centre for Experimental Pathogen Host Research (CEPHR), University College Dublin, Belfield, D04 V1W8 Dublin, Ireland; 2 Department of Infectious Diseases, St Vincent’s University Hospital, Elm Park, D04 TC63 Dublin, Ireland; 3 Center for Tropical Diseases and Global Health, Faculty of Medicine, Catholic University of Bukavu, Bukavu 73, Congo; 4 Division of Epidemiology and Biostatistics, Department of Global Health, Faculty of Medicine and Health Sciences, Stellenbosch University, Cape Town 7505, South Africa; 5 Cochrane South Africa, South African Medical Research Council, Cape Town 7505, South Africa; 6 Vaccine and Epidemics Research Group, Makerere University Lung Institute, Kampala P.O. Box 22412, Uganda; 7 Department of Medicine, Makerere University College of Health Sciences, Kampala P.O. Box 7072, Uganda; 8 Division of Infection and Global Health, School of Medicine, University of St Andrews, St Andrews KY16 9TF, UK; 9 Medical Research Council/Uganda Virus Research Institute and London School of Hygiene and Tropical Medicine Uganda Research Unit, Entebbe P.O. Box 49, Uganda

**Keywords:** mpox, MVA-BN vaccine, monkeypox virus, antibodies, B cells, T cells

## Abstract

The 2022 global mpox outbreak, caused by clade IIb of the monkeypox virus (MPXV), prompted emergency use authorisation of the Modified Vaccinia Ankara–Bavarian Nordic (MVA-BN) vaccine, previously approved for smallpox prevention. Understanding immune responses to the MVA-BN vaccine is critical to inform both current and future mpox vaccine policy, particularly amid reports of breakthrough infections in vaccinated persons, uncertainty about the durability of vaccine-induced protection, and the emergence of further outbreaks of mpox from different viral clades, including the clade I-driven public health emergency of international concern. MVA-BN elicits binding and neutralising antibody, memory B cells, and T cell responses. Immune responses vary by host factors, prior orthopoxvirus exposure, and dosing regimens. While seroconversion is generally robust, circulating antibody titres often wane rapidly, particularly in vaccinia-naïve and/or immunocompromised individuals, including people with HIV. Vaccine-induced neutralising antibody responses to MPXV are frequently lower than to vaccinia virus, and their role in protection remains ill-defined. In contrast, T cell responses appear more sustained and may support long-term immunity in the absence of persistent antibody titres. This narrative review synthesises current evidence on the immunogenicity and durability of MVA-BN vaccination, highlights challenges in assay interpretation, and outlines key research priorities, including the need to explore correlates of protection, booster strategies, and next-generation vaccine design.

## 1. Introduction

Monkeypox virus (MPXV), a zoonotic orthopoxvirus (OPXV), was first identified in 1958, with human cases reported in Central Africa from 1970 onward [1]. MPXV comprises two major genetic clades: clade I (formerly Congo Basin) and clade II (West African) [2]. Historically a zoonosis, with short chains of human to human transmission, mpox has undergone a major epidemiological shift with the 2022 global outbreak of clade IIb, characterised by sustained human to human transmission, predominantly among gay and bisexual men who have sex with men. This led to over 100,000 global cases, prompting the World Health Organisation (WHO) to declare a Public Health Emergency of International Concern (PHEIC) [3,4]. A second, separate PHEIC was declared in 2024 in response to a large outbreak of clade I mpox in Central Africa, marked by higher case fatality rates, particularly amongst children and women [5].

Due to high antigenic and genetic homology among OPXVs, including between MPXV and vaccinia virus (VACV), existing smallpox vaccines have been redirected for mpox prevention [6,7]. In the context of the PHEIC, the third-generation, non-replicating smallpox vaccine, Modified Vaccinia Ankara–Bavarian Nordic (MVA-BN) received emergency authorisation for mpox prophylaxis based on preclinical protection in non-human primate challenge models [8], immunogenicity data from phase I–III clinical trials [9,10,11], and observational evidence of reduced mpox susceptibility in previously smallpox-vaccinated individuals [12]. While a subsequent meta-analysis has estimated that two MVA-BN doses confer approximately 82% short-term protection against mpox infection [13], the real-world effectiveness, durability, and correlates of immunity remain ill-defined.

**Figure 1:**
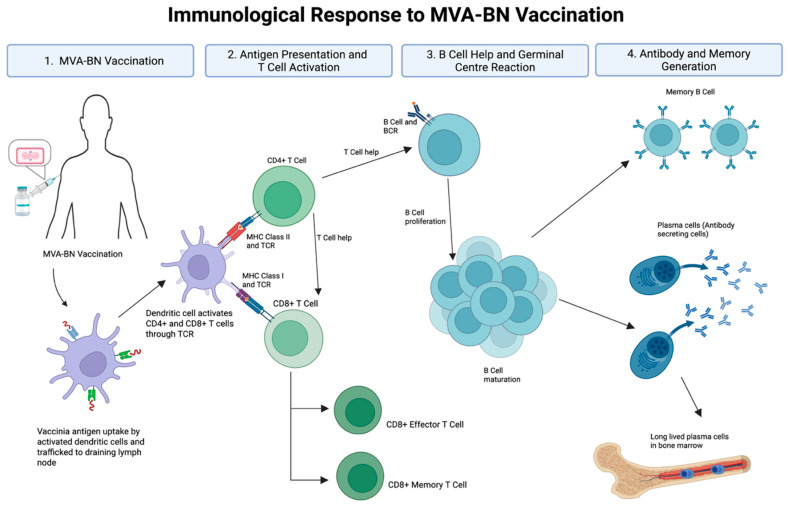
Immunological response to MVA-BN vaccination. Abbreviations: MVA-BN (Modified Vaccinia Ankara–Bavarian Nordic), TCR (T cell receptor), BCR (B cell receptor), MHC (major histocompatibility complex). Vaccinia antigen is taken up by dendritic cells and trafficked to draining lymph nodes, where CD4^+^ and CD8^+^ T cells are activated via MHC class II and class I pathways, respectively. Activated CD4^+^ T cells provide help to B cells, promoting proliferation, germinal centre formation, and affinity maturation. This leads to the generation of antibody-secreting plasma cells and long-lived memory B cells. CD8^+^ T cells differentiate into effector and memory subsets. Together, these processes underpin the humoral and cellular arms of MVA-BN vaccine-induced immunity. Development of durable, long-lived plasma cells remains an important goal for next-generation vaccine strategies. Created with BioRender.com (accessed on 23 April 2025).

Vaccine-mediated immune protection requires the induction of durable, functional immunity. MVA-BN induces both humoral and cellular responses, including the production of binding and neutralising antibodies and pathogen-specific memory B cells and T cells (Figure 1). However, several critical immunological questions remain. 1. How durable are antibody responses, particularly in VACV-naïve or immunocompromised individuals? 2. To what extent do T cell responses contribute to protection, and how long are they sustained? 3. Can memory B cell responses compensate for waning circulating antibody levels? 4. What is the optimal timing or need for booster vaccine doses? 5. And, critically, what immune parameters best predict protection against mpox infection? The emergence of this previously localised infection to a global geography offers an opportunity to find answers to many of these unknowns, offering a framework for addressing future infections of pandemic potential.

Answering these questions will not only help optimise the deployment of MVA-BN but also guide the design of next-generation OPXV vaccines, in particular to define correlates of protection (CoP). That vaccinated individuals continue to experience mpox infections raises concerns about the durability of vaccine-induced protection [14,15]. In addition, in the absence of a validated CoP, a more detailed understanding of the kinetics, quality, and breadth of vaccine-induced responses is needed, particularly in significant sub-populations, such as women, children, and people with HIV (PWH).

This review synthesizes current evidence on immune responses to MVA-BN vaccination, including the durability of humoral and cellular immunity, key determinants of immunogenicity (Table 1), and methodological limitations of serological assays. We also discuss gaps in mucosal and memory B cell data, emerging vaccine strategies to enhance protection, including delayed boosting and mRNA platforms, and how immune profiling tools can help define correlates of immunity in the mpox vaccine landscape.

## 2. MVA-BN Vaccine

Vaccinia-targeting vaccines have been more widely used for human immunisation than any other vaccine platform. For almost two centuries, VACV-based vaccines were employed to provide cross-protection against smallpox, until the disease was eradicated in the 1970s [34]. Although first-generation (DryVax^®^) and second-generation (ACAM2000^®^) replication-competent smallpox vaccinations were successful, they were associated with severe side effects, including eczema vaccinatum, progressive clinical vaccinia infection, myopericarditis, and post-vaccination encephalitis, particularly in individuals with underlying immunodeficiencies, skin disorders, or pregnant individuals [35,36].

The third-generation MVA-BN vaccine (marketed as Imvamune^®^, Jynneos^®^, and Imvanex^®^) was developed to address safety concerns of early smallpox vaccines. It is a live, attenuated vaccine, rendered replication-deficient in human cells through serial passage in avian cells, resulting in a 14% genomic deletion compared to the parental VACV genome [37,38,39].

Evidence from phase I/II clinical trials demonstrates that MVA-BN elicits humoral and cellular immune responses similar to previous-generation smallpox vaccines [40,41,42]. In a phase III, randomised controlled trial (RCT), a two-dose MVA-BN regimen followed by a boost with ACAM2000^®^ was non-inferior to ACAM2000^®^ alone in terms of seroconversion and peak neutralising antibody (nAb) titres and associated with fewer local adverse events [11].

Placing these findings in context, since smallpox eradication precluded field efficacy trials, clinical evaluation of smallpox vaccines, including MVA-BN, has relied exclusively on surrogate immunological endpoints, such as seroconversion rates and nAb titres. While these markers provided regulatory justification for vaccine approval, their relevance to real-world protection against smallpox remains uncertain, even more so for protection against other OPXVs, such as mpox. The reliance on cross-protective immunity, derived largely from VACV-based antigens, underscores a critical gap in the response to mpox and highlights the absence of a validated, mpox-specific CoP. This limitation has become increasingly salient in the context of breakthrough infections, highlighting the need for biomarkers that accurately reflect protective immunity against MPXV rather than reliance on inferred OPXV cross-reactivity immunity.

While no RCTs had assessed MVA-BN efficacy against mpox prior to the 2022 outbreak, observational data from historical outbreaks suggested that prior smallpox vaccination reduced susceptibility to mpox, with lower attack rates observed in vaccinated individuals [43,44]. Preclinical studies in non-human primates later provided critical mechanistic insights. MVA-BN vaccination induced both binding and neutralising antibodies targeting both intracellular mature virions (IMVs) and extracellular enveloped virions (EEVs), along with robust T cell responses [45,46]. These immune responses mirrored those elicited by first-generation vaccines and conferred protection against MPXV challenge, including reduced viraemia and attenuated clinical disease [45].

On the basis of this evidence, the MVA-BN vaccine was granted emergency authorisation for use as both mpox pre- and post-exposure prophylaxis during the mpox PHEIC. The standard regimen for smallpox vaccine-naïve individuals is two doses of 0.5 mL MVA-BN subcutaneously 28 days apart and a single dose in individuals with a history of previous smallpox vaccination. In light of global vaccine shortages, a fractionated intradermal dose regimen (one-fifth of the standard dose) was adopted on a pragmatic basis at the start of the outbreak in 2022 and demonstrated to be immunologically non-inferior, enabling expanded access [47]. These data established MVA-BN as a viable platform for mpox control, but its real-world effectiveness, particularly the magnitude and persistence of mpox-specific immune responses, remains incompletely characterised.

## 3. Challenges in Serologic Assays for Antibody Evaluation

Accurate interpretation of vaccine-induced immune responses relies heavily on the assays used to detect them. Evaluating antibody responses after MVA-BN vaccination presents several technical challenges, largely due to the complexity of OPXV antigen cross-reactivity. The substantial heterogeneity in design, implementation, and interpretation of different serological assays further complicates cross-study comparisons. Antibody titres have been measured using various platforms, including electrochemiluminescence immunoassays, enzyme-linked immunosorbent assays (ELISAs), and Luminex^®^ bead-based systems, which differ in their dynamic range, sensitivity, and background reactivity [48].

Serological assays vary significantly in their antigenic epitope targets, introducing substantial heterogeneity into immune response assessment. Some assay platforms employ whole-virus lysates, while others use defined OPXV antigens derived from either VACV or MPXV. Although many immunodominant antigens are conserved across these species, this homology is not uniform. For example, homologous antigens VACV B5 and MPXV B6R are highly conserved across species, whereas others diverge considerably [49]. Consequently, assays may capture antibodies elicited by MVA-BN vaccination, prior smallpox vaccination, or natural mpox infection, often without clear distinction. Notably, an electrochemiluminescence assay uses a combination of three antigens, MPXV B6R, MPXV A27L, and VACV B5, to quantify antibodies to both infection and vaccination and to distinguish between them with high sensitivity and specificity [16].

In addition, antigen selection may reflect different stages of the OPXV replication cycle, including intracellular mature virions (IMVs) and extracellular enveloped virions (EEVs). This is immunologically significant. IMV antigens, such as MPXV A29L and A27L, are primarily involved in early infection and containment at the site of inoculation, whereas EEV antigens, such as MPXV B6R and MPXV A35R, mediate viral dissemination and are essential for systemic spread [50,51,52]. Table 2 provides an overview of OPXV antigens used in immunogenicity studies. In a cohort study that evaluated 13 OPXV antigens, anti-VACV B5 IgG provided the best combined sensitivity and specificity for detecting prior antigen exposure and quantifying vaccine-induced IgG [16]. However, many OPXV antigens remain incompletely characterised, and antigenic divergence between VACV and MPXV adds further uncertainty [49,53]. As a result, variability in antigen source (VACV vs. MPXV) and stage specificity (IMV vs. EEV) introduces potential variability and inconsistencies in assay sensitivity, specificity, and functional relevance between serological assays [16,54]. 

## 4. Seropositivity Thresholds for Antibody Responses

Establishing reliable seropositivity thresholds for protection against infection or severe disease is essential for interpreting antibody responses following MVA-BN vaccination. In most studies, these thresholds are derived from historical control cohorts, yet the immune status of these individuals is often uncertain, particularly given the cessation of national immunisation programmes against smallpox, the increasing use of immunosuppressive medications, and the prevalence of secondary immunosuppression from conditions such as malignancy and HIV in specific sub-populations at risk [58]. Data from electrochemiluminescence and Luminex^®^-based assays targeting multiple OPXV antigens have shown that even well-curated presumed-negative controls exhibit low-level background immune responses to viral antigens, complicating the ability of the serological assays to discriminate between true positives and false positives [16,54]. Without an international reference standard or WHO-endorsed mpox reference serum, thresholds remain assay-specific, are not cross-comparable, and most importantly, may not reliably correspond to functional immune protection. As such, antibody titres must be interpreted in the context of assay design, antigen target, and timing of samples relative to exposure or vaccination.

## 5. Kinetics and Magnitude of Binding Antibody Responses

The MVA-BN vaccine elicits a rapid binding antibody response, with seroconversion rates exceeding 90% by two weeks following the second dose [11,16,17,18]. Peak binding IgG titres are typically observed two weeks post-second dose vaccine, with seropositivity sustained through the first 90 days post-vaccination, consistent with an early plasmablast-driven response [16,19]. However, titres substantially decline thereafter, with multiple studies documenting waning of IgG titres at 6 to 12 months post-vaccination [16,17,20,21]. One modelling study estimated an antibody half-life of approximately 108 days [20]. The route of administration appears to exert a limited influence on initial immunogenicity. Fractional intradermal administration has been shown to be non-inferior to full-dose subcutaneous regimens up to 180 days post-vaccination, offering a feasible strategy for dose-sparing, mass vaccination as part of outbreak response efforts [20,47].

The magnitude and kinetics of response are modulated by prior OPXV exposure, the number of vaccine doses administered, and host factors. In individuals previously vaccinated against smallpox, a single MVA-BN dose triggers a rapid recall of memory B cell responses, with seroconversion and antibody rises observed within 2–4 weeks [9,23]. In contrast, VACV-naïve individuals require two doses to reach similar peak titres. Nevertheless, studies consistently report that after completing the full recommended schedule (one dose for primed, two doses for naïve), peak antibody titres converge between groups [18]. This convergence in final antibody magnitude supports the efficacy of the two-dose schedule in VACV-naïve individuals and underscores the importance of adhering to the full regimen, particularly in immunocompromised populations, such as PWH, where immune priming status may be blunted [23,24].

Despite comparable peak titres, the durability of immune responses remains a key limitation. VACV-naïve individuals frequently experience a rapid decline in antibody titres, approaching baseline within 5 to 7 months post-vaccination. In contrast, VACV-primed individuals tend to maintain higher titres, indicating more durable secondary responses [22]. Recent modelling suggests that extending the interval between the first and second MVA-BN doses, from the standard 28 days to two years, could potentially enhance both peak and sustained antibody levels, with predicted three- to four-fold higher peak titres post-second dose and a fourteen-fold increase in antibody levels at one year post-second dose [13]. These findings support further exploration of alternative prime-boost intervals to optimise long-term protection and are not merely of academic interest; they represent potentially critical, resource-sparing strategies to maximise vaccine impact and achieve equity in mpox-endemic regions of Africa where supply is constrained.

## 6. Durability of Humoral Immunity

The global eradication of smallpox in the 1970s created a rare immunological context: a population cohort primed with previous-generation live smallpox vaccines yet no longer exposed to circulating OPXVs. This offered a unique opportunity to evaluate the long-term durability of vaccine-induced immunity in the absence of natural boosting. While immunological memory to previous-generation live smallpox vaccines has been demonstrated to persist for decades [55,59], the long-term durability of immunity induced by the third-generation, non-replicating MVA-BN vaccine remains less certain.

Emerging data suggest that antibody responses to MVA-BN may be less durable than those elicited by replicating smallpox vaccines. In a cohort of healthcare workers in the Democratic Republic of Congo (DRC), OPXV-specific IgG titres measured two years after a two-dose MVA-BN regimen were not significantly different from baseline, regardless of prior smallpox vaccination status [18]. Similarly, a longitudinal cohort study in Ireland showed a significant decline in antibody titres over two years, with modelling predicting that mean titres would fall below the seropositivity threshold approximately 15.5 (95% CI: 13.0–19.5) months post-vaccination. By two years, the majority of vaccine recipients no longer demonstrated detectable seropositivity to anti-VACV B5 IgG [25]. The non-replicating MVA-BN vaccine may generate less durable immune responses compared to live vaccines, potentially due to differences in the magnitude or duration of antigen presentation and immune stimulation, which may be insufficient to induce or maintain long-lived plasma cells in the bone marrow [60,61].

Taken together, the rapid waning of circulating antibodies, limited cross-neutralisation against MPXV, and poor mucosal responses strongly indicate that while MVA-BN provides effective short-term protection, it is unlikely to confer long-term sterilising immunity in most VACV-naïve individuals. This positions cellular and memory responses not as a potential offset but as the primary mechanism for long-term protection against severe disease and underscores the urgent need for research into booster strategies to maintain high levels of protective immunity.

## 7. Determinants of Antibody Responses

Among the host factors determining response to vaccination, immunogenicity in children, women, and PWH has been the subject of particular focus, given both its disproportionate burden of severe mpox infection and mortality [62] and, in the case of PWH, historical contraindications to vaccination with live replicating vaccines [63]. MVA-BN has demonstrated a favourable safety profile in PWH, including those with advanced HIV [26,27]. However, the reduced peak titres and more rapid waning of humoral responses in this population are well documented. In a phase II RCT, PWH with a history of advanced HIV (nadir CD4+ T cell < 200 cells/mm^3^) mounted significantly lower peak VACV IgG titres than people without HIV, and titres declined more sharply over six months [26]. These findings are consistent across studies and timepoints, with lower binding and nAb titres, faster decay rates, and a correlation between lower CD4+ T cell counts and lower antibody levels [20,27,28]. This impaired humoral response remains ill-defined but likely reflects broader immunological deficits that are well described in PWH, including residual immune activation, disrupted germinal centre architecture, and premature B cell senescence [64].

In addition to immunological factors, such as HIV, emerging evidence suggests that biological sex may modulate vaccine-induced antibody responses. One meta-analysis reported higher antibody titres in males following MVA vaccination, which may reflect genetic or immunoregulatory differences [65]. Although these findings are limited in scope, these data gaps are particularly concerning given that women and children account for a substantial proportion of individuals affected by the ongoing clade I PHEIC in Central Africa.

The absence of robust data on the safety and efficacy of the MVA-BN vaccine in pregnancy is concerning, as mpox in pregnancy carries significant morbidity and mortality risks, including congenital infection and pregnancy [66,67]. Current guidance advises avoiding the MVA-BN vaccine in pregnancy unless the possible benefits in terms of preventing mpox outweigh any potential risk of the vaccine. This highlights the urgent need for immunogenicity and durability studies in underrepresented populations, including women, children, and affected communities in Central Africa, which have been historically underrepresented in immunological studies [68]. Currently, two trials are underway in Central Africa: PregInPoxVac (NCT06844487), a clinical trial in DRC that will assess the safety and immunogenicity of the MVA-BN vaccine in pregnant women, newborns, and young infants, and MpoxVax AFRIVAC, a clinical trial across the DRC, Uganda, and Tanzania that will determine the relative immunogenicity of mpox vaccination in immunosuppressed individuals and women as well as compare the durability of immune responses in different populations (European versus African). This gap in knowledge is not an oversight but a critical failure of the global research response, leaving clinicians and public health officials without essential data for the populations, women, and children bearing a significant burden of the ongoing clade I outbreak in Central Africa. Furthermore, the genetic divergence between the clade I virus driving the current PHEIC and the clade IIb virus from the 2022 outbreak raises a critical question about the extent to which MVA-BN-induced immunity remains protective. Prospective efficacy studies in clade I-endemic regions are urgently needed to assess and refine current vaccination strategies.

## 8. Mucosal Antibody Responses and Site-Specific Immunity

Mucosal immunity, in theory, could play an important role in preventing mpox acquisition, given that transmission often occurs through contact with mucosal surfaces, such as during sexual activity. Effective local immune responses, including dendritic cell activation and antibody secretion at mucosal sites, are particularly important in PWH, who may have impaired mucosal immune function [69]. Mucosal immunity remains insufficiently characterised following MVA-BN vaccination. A recent cohort study detected IgA to VACV and MPXV antigens using ELISA in saliva but not in rectal mucosa up to six months post-vaccination [29]. This may reflect inefficient homing of vaccine-induced B cells to mucosal tissues, limited local antigen presentation, insufficient secretion of antibodies in specific sites, or insufficient priming of mucosal-associated lymphoid structures. While initial systemic IgG and IgA responses are robust, the absence of rectal mucosal antibodies raises concerns about incomplete protection at vulnerable portals of mpox virus entry, which may contribute to ongoing transmission, despite vaccination. An urgent priority is to characterise MVA-BN-induced mucosal responses in detail and prospectively correlate them with breakthrough infection by exposure route. If suboptimal mucosal immunity is confirmed and linked to increased risk, targeted mucosal adjuvant strategies should be prioritised in next-generation vaccine research.

Taken together, these findings underscore that while MVA-BN induces rapid and robust early systemic antibody responses, the durability of antibody levels is limited, particularly in VACV-naïve or immunocompromised individuals. Intradermal route of administration and delayed boosting may optimise responses in resource-limited settings. However, the observed gaps in our understanding of long-term immunity, mucosal protection, and responses in vulnerable sub-populations highlight critical priorities for future research.

## 9. Neutralising Antibodies

While circulating antibodies offer a broad indication of humoral immune activation post-vaccination, only a subset of these antibodies confers functional protection. Most serological assays assess binding antibodies targeting either one or a small, limited set of viral antigens rather than the full viral proteome, which may underestimate the breadth or functional relevance of the humoral response. Measurement of nAbs, which block viral entry and replication, or viral neutralising activity, which measures broader host immunity by measuring the ability of a host plasma sample to prevent mpox infection of cells in vitro, are considered a more direct CoP.

Multiple methods are used to quantify viral neutralising activity, including live virus neutralisation tests, such as plaque reduction neutralisation testing (PRNT), considered the gold standard, pseudovirus-based assays, and surrogate virus neutralisation tests. These platforms differ in sensitivity, complexity, and throughput, complicating inter-study comparisons [48]. In addition, the lack of reference reagents, such as reference viruses or standardised viral antigens, provided as international units for use in assays, further limits the utility of viral neutralisation assays as universal biomarkers of immunity.

Longitudinal data consistently show that MVA-BN-induced nAb responses wane over time, particularly in VACV-naïve individuals. In an observational study using plaque reduction neutralisation testing (PRNT), the majority of VACV-primed individuals retained MPXV-neutralising activity one year post-vaccination, whereas only one-third of VACV-naïve individuals did so, and at substantially lower titres [21]. Similar patterns were observed in other cohorts; nAb titres against both VACV and MPXV peaked within two weeks after the second MVA-BN dose and declined significantly by two years, with PRNT50 titres correlating only moderately with total IgG levels [18]. Longitudinal data from a DRC cohort in 2022 showed a similar pattern: nAbs against both VACV and MPXV peaked by two weeks post-second dose and declined significantly by two years, with PRNT50 titres correlating moderately with IgG levels [18]. In vitro, neutralisation against MPXV is consistently lower than against VACV across studies of the MVA-BN vaccine [18,29,30], likely reflecting preferential recall of conserved VACV epitopes in previously vaccinated individuals. This suggests limited induction of de novo MPXV-specific responses and may help explain breakthrough infections among MVA-BN recipients [15].

## 10. Fc-Mediated Effector Functions

Beyond direct neutralisation, antibodies can engage Fc-mediated effector functions, in which the fragment crystallisable (Fc) region interacts with Fcγ receptors or complement proteins to trigger downstream immune mechanisms. These include antibody-dependent cellular cytotoxicity (ADCC), antibody-dependent cellular phagocytosis (ADCP), and antibody-dependent complement deposition (ADCD). Such mechanisms can facilitate clearance of infected cells and enhance viral control, particularly when nAb titres are low. In OPXV research, ADCD has been shown to enhance cross-neutralisation between VACV and MPXV [29], underscoring the potential importance of Fc-dependent pathways. Evidence from the SARS-CoV-2 vaccination shows how non-nAbs, which are unable to block viral entry, can still confer protection via Fc-mediated mechanisms. Moreover, Fc-effector activity endures long after peak nAbs have waned, suggesting an important role in sustained protection over time [70]. Although data for MVA-BN are limited, incorporating these assessments into functional immune assays could provide a more complete understanding of vaccine-induced immunity.

## 11. B Cell Memory

Although circulating antibody levels wane over time, a robust memory B cell compartment can compensate by enabling rapid antibody production upon re-exposure. In support of this, individuals who received a booster dose two years after initial MVA-BN vaccination exhibited a rapid rise in nAb titres, surpassing levels observed after primary vaccination [23]. This anamnestic response provides strong functional evidence for the presence of long-lived memory B cells and suggests that MVA-BN vaccination can induce durable immunological memory, even in the absence of sustained high-titre circulating antibodies.

However, direct mechanistic evidence for B cell memory remains limited. An early study reported minimal increases in circulating plasmablasts, somatic hypermutation, or antibody repertoire diversification two weeks post-MVA-BN vaccination [32]. Although mRNA vaccines, such as those targeting SARS-CoV-2, have been shown to induce prolonged germinal centre activity [71], this may not be a universal feature of all non-replicating platforms. Despite its initial apparent immunogenicity, MVA-BN may generate limited germinal centre-derived affinity maturation in some settings, possibly due to differences in antigen persistence or innate immune sensing.

Germinal centre activity is essential for generating high-affinity, long-lived memory B cells. An observational study found that MPXV H3 and A35 antigen-specific memory B cells were detectable at one year post-vaccination, but at significantly lower frequencies than in individuals who had recovered from natural mpox infection [22]. Moreover, VACV-naïve individuals vaccinated with MVA-BN exhibited lower binding avidity for MPXV antigens compared to those with prior smallpox vaccination, consistent with incomplete affinity maturation. This suggests that, while memory B cells are inducible by MVA-BN, their quality and magnitude may be limited in VACV-naïve populations. In these studies, memory B cell responses were not stratified by HIV status; therefore, the impact of HIV infection on the quality and durability of B cell immunity following MVA-BN vaccination could not be assessed. Taken together, these findings raise concerns about the strength and durability of B cell memory post-MVA-BN and highlight the need for better mechanistic studies.

## 12. T Cell Responses

In contrast to humoral responses, vaccine-induced T cell responses appear more durable and may contribute to sustained protection by limiting viral replication and supporting secondary antibody responses, particularly when circulating antibodies decline. Although data on T cell immunity following MVA-BN vaccination remain limited, emerging evidence supports a key role for both CD4^+^ and CD8^+^ T cells in long-term protection. In non-human primate models, MVA-based vaccines elicit robust CD4^+^ and CD8^+^ T cell responses comparable to those induced by replicating first-generation smallpox vaccines [45].

Several clinical studies have demonstrated rapid induction of MVA-BN-specific T cell responses. Using IFN-γ ELISpot, activation-induced marker assays, and intracellular cytokine staining, both CD4^+^ and CD8^+^ T cell responses were detectable within two weeks of the first dose, marked by the upregulation of OX-40, CD69, and CD137 and the production of IFN-γ, indicating functional activation consistent with the induction of antigen-specific memory T cell responses, although formal subset delineation was not performed [19,29]. Notably, the magnitude of T cell responses did not correlate with seroconversion, suggesting independent regulation of humoral and cellular arms of immunity [19]. Another study using IFNγ ELISpot showed that MVA-BN vaccination elicited robust effector memory T cell populations up to one month post-vaccination. MPXV-specific CD8^+^ T cells displayed a CD45RA^+^CCR7^−^ effector memory phenotype, with low expression of senescence marker CD57 and preserved CD27, indicative of an early/intermediate differentiation state [72].

An observational cohort study directly compared T cell responses following vaccination and infection. Vaccinated individuals developed detectable CD4^+^ central memory and effector memory responses by 28 days post-vaccination, but CD4^+^ central memory frequencies and IL-2 production were reduced relative to convalescent individuals. Similarly, CD4^+^ effector memory was present at comparable frequencies, but with lower functional capacity and diminished IL-2 production. Effector subsets, including CD45RA^+^CCR7^−^ TEMRA cells, remained low after vaccination and did not expand to levels seen after infection [73]. These findings suggest that while MVA-BN is capable of generating functional antigen-experienced memory T cells, infection induces a broader and more functionally robust memory compartment, which may explain the more robust immune response observed post-infection than vaccination [25].

T cell responses were observed against both conserved OPXV epitopes and MPXV-specific antigens, with one study demonstrating that over 80% of CD4^+^ and CD8^+^ T cell epitopes derived from VACV are conserved in MPXV, and many are encoded by early viral genes retained in the MVA-BN genome [74]. These conserved epitopes elicited strong responses in Dryvax^®^ vaccinees, including cytotoxic CD4^+^ subsets, supporting the likelihood of MPXV cross-reactive T cell immunity, even in the absence of MPXV-targeted antibodies.

Importantly, while MPXV-nAbs decline significantly over time, longitudinal studies show that MVA-BN-induced T cell responses persist at six and twelve months post-vaccination [35]. This durability, coupled with emerging reports of breakthrough infections in individuals with high antibody titres [75], underscores the likely contribution of T cell immunity as a complementary arm of protection, particularly in high-risk or immunocompromised populations, such as PWH and children.

In PWH, T cell responses following MVA-BN appear preserved, though response kinetics and boosting differ by CD4^+^ T cell count and vaccination strategy. One study demonstrated that CD4^+^ responses increased after the first dose with minimal further boosting, while CD8^+^ responses rose more substantially after the second dose [31]. A two-dose regimen yielded stronger T cell responses than a single dose, particularly in PWH [24]. A cohort study found that PWH with CD4^+^ counts ≤ 500/mm^3^ mounted weaker T cell responses and required two doses for comparable immunity, suggesting that more advanced immunodeficiency may impair vaccine-induced T cell responses [33]. Intradermal delivery was more immunogenic than subcutaneous administration, generating Th1-skewed and polyfunctional responses, particularly in individuals with higher CD4^+^ counts [33]. This may reflect the skin’s high density of antigen-presenting cells and supports further exploration of alternative delivery strategies to optimise T cell responses.

## 13. Correlates of Protection

Identifying reliable correlates of protection (CoPs) is a critical unmet need for mpox vaccine development and policy. Neutralising antibody (nAb) titres have been proposed as a candidate CoP based on robust data from non-human primate models, where nAbs limit viral entry and replication, reducing lesion count and systemic dissemination [76]. nAbs are well-established correlates for several licensed vaccines, including previous-generation smallpox vaccines, and offer a biologically plausible link to protection [77,78].

However, unlike serological assays, measuring CoPs through gold-standard live-virus neutralisation assays poses substantial practical limitations. These assays are resource-intensive, technically demanding, and require biosafety level 3 facilities. Such constraints limit their scalability, particularly in large trials and in low-resource settings, such as the DRC, which is bearing the brunt of the current ongoing clade I mpox outbreak. To circumvent these barriers, VACV-specific binding IgGs have been explored as surrogate markers. These titres correlate with MPXV-neutralising activity and vaccine effectiveness in some studies [13,18].

A validated CoP for mpox remains undefined. A key uncertainty is whether peak post-vaccine antibody titres can reliably predict durable protection, particularly in light of antibody waning. This challenge is underscored by growing reports of breakthrough infections among vaccinated individuals [14,15], including cases occurring despite high circulating antibody titres [75]. These observations suggest that humoral markers alone may be an insufficient correlation of protection. Rather than relying on a single metric, a comprehensive immunological profile that integrates T cell responses and functional B cell memory may be necessary to accurately assess vaccine-induced protection.

To address this complexity, systems vaccinology offers a promising approach. By integrating high-dimensional immune profiling with longitudinal clinical data, this strategy can uncover predictive immune signatures. This approach has previously revealed predictive signatures of vaccine-induced durability and effectiveness against COVID-19 and influenza [79,80]. Applying such methods in mpox vaccine trials could reveal composite immunological profiles that may better reflect protection than any single marker alone.

Establishing a CoP for mpox will require coordinated, longitudinal studies incorporating diverse populations, particularly those at highest risk of severe disease, in settings of high risk of transmission or suboptimal vaccine response. While a systems immunology framework that integrates cellular, humoral, and mucosal compartments may uncover novel insights and predictive signatures, the ultimate goal should remain to identify CoPs that are simple, reliable, and practical to measure in clinical and public health settings.

## 14. Role of Booster Vaccines

The current recommended schedule for MVA-BN is two doses administered 28 days apart; however, the rising incidence of breakthrough mpox cases in MVA-BN-vaccinated individuals has renewed attention on the potential need to sustain long-term protection through booster strategies [14,15]. Data from a phase II, randomised, non-inferiority trial and its follow-up open-label booster study conducted in Europe between 2006 and 2009 demonstrated that individuals primed with one (*n* = 77) or two (*n* = 75) MVA-BN doses mounted strong and rapid anamnestic responses following a single booster administered two years later, with nAb titres post-booster exceeding those observed after primary vaccination [23]. This rapid and robust immune recall response likely reflects durable memory B cell generation. These findings support the use of booster doses to reverse waning immunity, particularly in high-risk populations. Although PWH were not directly evaluated in this study, evidence suggests they experience faster antibody waning following MVA-BN [25,26], indicating that this group may derive particular benefit from booster vaccination and should be prioritised in future booster trials.

Building on these findings, a recent modelling study compared standard dosing (two doses, administered 28 days apart), delayed dosing (second dose at 730 days), and the addition of a third “booster” at 730 days [13]. The analysis suggested that administering a third dose two years after the standard two-dose regimen increased antibody titres at one year post-booster compared with titres measured one year after the initial two-dose course. However, importantly, the third dose did not confer additional long-term durability beyond that achieved by delaying the second dose to two years, suggesting that dose timing may be as critical as the absolute number of doses in determining the longevity of protection [13].

Despite these insights, no clinical trials have directly evaluated the optimal timing of booster vaccination in humans, and evidence from real-world settings remains sparse, especially among immunocompromised groups, such as PWH. In the absence of an established CoP, both the ideal interval and relevant population targets for booster administration remain ill-defined. Any discussion of booster implementation must consider the broader global context. While high-resource countries may consider additional booster vaccination to sustain protection, access to MVA-BN for initial vaccination remains limited in many resource-limited countries, especially in Africa, where mpox is endemic and the PHEIC continues. The value of directing limited vaccine supplies toward booster campaigns in low-incidence settings must be weighed against the urgent unmet needs in endemic regions, where the epidemic is being sustained, in large part due to a lack of resources.

## 15. Future Directions

The development of next-generation vaccines offers promising opportunities. As of November 2024, twenty candidate mpox vaccines are in development, most of which are nucleic acid vaccine platforms [81]. mRNA-based platforms, which demonstrated durability in SARS-CoV-2 vaccination [82,83], represent the majority of candidates under investigation for mpox. However, only three (mRNA-1769, BNT166a, and BNT166c) have progressed to the clinical phase of vaccine research. In non-human primate models, mRNA vaccination elicited higher binding and neutralising antibody responses than MVA-BN and conferred protection against an MPXV challenge [84,85]. While these findings are encouraging, validation in human cohorts, particularly in VACV-naïve and immunocompromised populations, is essential.

## 16. Conclusions

Current evidence indicates that while MVA-BN vaccination induces both antibody and T cell responses against OPXV, the durability of MPXV-specific humoral immunity is limited, particularly in VACV-naïve individuals and PWH. Although anamnestic responses following booster vaccination suggest preserved immunological memory, direct characterisation of B cell memory at the cellular level remains limited. In contrast, T cell responses appear more stable over time and may provide complementary protection as antibody titres wane, though their precise contribution to clinical protection remains to be fully delineated.

As mpox remains a PHEIC, defining an immunological CoP is an urgent research priority. The absence of a validated correlate impedes interpretation of immunogenicity data and complicates the development of evidence-based vaccine strategies. Advances in vaccine technology, such as systems vaccinology, scalable serological platforms, and the use of longitudinal cohort studies, can help accelerate vaccine development, CoP identification, and implementation [79,86,87]. Applying these tools to mpox will be critical for developing durable and equitable protection, particularly in underserved populations and endemic regions. Building on this infrastructure offers a pathway to refine current vaccines, design next-generation platforms, and inform policy decisions for mpox and future OPXV threats.

## Figures and Tables

**Table 1:** Summary of key immunological outcomes following MVA-BN vaccination across clinical studies.

	Study (Ref)	Study Type	Vaccine Regimen	Study Population	Location	Timepoints Analysed	Immunoassay	Key Findings
Antibody	Greenberg et al., PloS One, 2016 [9]	Randomised, double-blind, placebo-controlled Phase II trial	2× MVA vs. 1× MVA after placebo	VACV-experienced adults (*n* = 120)	USA	Weeks 0, 2, 6; month 6	ELISA, PRNT	High seroconversion IgG rates and nAb post-dose 2. GMTs were higher in the two-dose group. A single dose elicited a strong memory response.
	Pittman et al., NEJM, 2019 [11]	Phase III RCT	2× MVA + 1× ACAM2000 vs. 1× ACAM2000	Healthy, VACV-naïve adults (*n* = 440)	USA, South Korea	Weeks 1, 2, 4, 6, 8, 12	ELISA, PRNT	Similar VACV IgG seroconversion rates (MVA-BN 90.8% vs. ACAM2000 91.8%). MVA was noninferior to ACAM2000 for peak nAb titres (GMT 153.5 vs. 79.3).
	Byrne et al., eBioMedicine, 2025 [16]	Observational Cohort Study	2× MVA	Adults (*n* = 167), including PWH	Ireland	Up to 1 year	ECL Assay	High VACV IgG seroconversion rates within the first 90 days, significant waning of IgG titres up to 1 year.
	Collier et al., JAMA, 2024 [17]	Observational Cohort Study	1 or 2× MVA	Adults (*n* = 45)	USA	Weeks 0, 3; months 3, 6, 9, 12	ELISA, flow cytometry-based live virus neutralisation assay	VACV IgG peaked at 3 weeks and then declined significantly by 12 months. NAb titres were low for 3 months.
	Priyamvada et al., Vaccine, 2022 [18]	Observational Cohort Study	2× MVA	Healthcare workers (*n* = 999), stratified by prior VACV vaccination	DRC	Weeks 0, 2, 4, 6; months 6, 12, 18, 24	PRNT, ELISA	Both naïve and previously vaccinated participants mounted robust VACV- and MPXV-nAbs, peaking at week 6. IgG titres waned by 2 years, but seropositivity was retained in most.
	Drennan et al., Lancet Microbe, 2025 [19]	Observational Cohort Study	2× MVA	Adults (*n* = 34), HIV-negative	United Kingdom	Days 0, 1, 14, 28 (post-dose 1), 28, 90 (post-dose 2)	Luminex	89% seropositivity by day 90 post-dose 2, though <50% seroconverted by day 28 post-dose 1.
	Kottkamp et al., NEJM, 2023 [20]	Observational Cohort Study	1 or 2× MVA	Adults (*n* = 145), 24% with HIV, 20% VACV-primed	USA	Up to 3 months post-vaccine	ELISA	IgG titres declined over time (half-life ~108 days). Titres were similar regardless of route (ID vs. SC), dose interval, or HIV status. Prior VACV vaccination conferred greater antibody durability.
	Matusali et al., Journal of Infection, 2024 [21]	Observational Cohort Study	1 or 2× MVA	Adults (*n* = 50), 42% PWH, 50% VACV-primed	Italy	Months 0, 1, 6, 12	ELISA, pseudovirus-based neutralisation assay	In primed individuals, 72% remained seropositive at 12 months. MPXV-neutralising antibodies declined over time, detectable in 32% of non-primed individuals at 12 months. No difference by HIV status.
	Oom et al., Journal of Virology, 2025 [22]	Observational Cohort Study	2× MVA	Adults (*n* = 159), including PWH, 31% prior VACV vaccination	USA	Weeks 0, 3; Months 3, 9	ELISA, Luminex, live-virus microneutralisation	nABs wane by 6 months in VACV-naïve. Both IgG and nAb were more durable in those with prior VACV vaccination.
	Ilchmann et al., JID, 2022 [23]	Phase II Randomised, Placebo-Controlled (With Follow-Up Booster Study)	1× or 2× MVA, or placebo; single MVA booster at 2 years	Adults (*n* = 745), adults (*n* = 152) in booster study	Germany	Weeks 0, 2, 4, 6, 8, 30, and 2 years; post-booster at weeks 1, 2, 4, and month 6	PRNT, ELISA	Boosting 2 years later led to rapid increases in nAbs. Total antibodies post-booster were highest in the 2× MVA group.
	Mazzotta et al., eClinicalMedicine, 2024 [24]	Observational Cohort Study	1× MVA VACV naïve, 2× MVA VACV experienced	Adults (*n* = 164), including 46% PWH	Italy	Months 0, 1	ELISA, PRNT	No difference in IgG between 1 dose vs. 2 doses overall, but 2 doses were more effective at eliciting nAbs in PWH.
	Byrne et al., 2025 [OFID, 2025] [25]	Observational Cohort Study	2× MVA	Adults post-vaccination (*n* = 122), 25% PWH	Ireland	Up to 2 years	ECL assay	Mean IgG titres declined below the seropositivity threshold by 15.5 months. At 2 years, 32% remained seropositive. PWH had significantly lower odds of sustained seropositivity at 2 years.
	Overton et al., Vaccine, 2020 [26]	Randomised Phase II Trial	2× MVA-BN (standard), 2× double dose, or 2× standard + booster at week 12	PWH with a history of advanced HIV (*n* = 87)	USA	Weeks 0, 4, 6, 12, 14, 30, 56	PRNT, ELISA	Double dose offered no immunogenicity advantage over standard. The booster improved peak and sustained nAbs.
	Greenberg et al., JID, 2013 [27]	Phase I/II Trial	2× MVA-BN in VACV-naïve, 1× in VACV-experienced	Adults (*n* = 151), including PWH (*n* = 91)	USA	Days 0, 14, 42; week 8; month 6	PRNT, ELISA	NaB and IgG responses were comparable across groups. Boosting in VACV-experienced participants showed strong anamnestic responses. GMTs were lower in PWH.
	Moschetta et al., Lancet Infect Dis, 2023 [28]	Observational Cohort Study	2× MVA	Adults (*n* = 85), including PWH	Italy	Month 6	PRNT	12% had no detectable nAb. PWH had higher odds of low titres.
	Crandell et al., Lancet Microbe, 2025 [29]	Observational Cohort Study	2× MVA	Adults (*n* = 111), including VACV-experienced (*n* = 36)	USA, Brazil, Portugal	Up to 11 months	ELISA, PRNT	MVA induced strong anti-VACV responses but limited cross-neutralisation against MPXV. MPXV nAbs waned to baseline within 6–11 months. Boosting with MVA in VACV-experienced enhanced breadth and durability.
	Zaeck et al., Nat Med, 2023 [30]	Observational Cohort Study	2× MVA	Adults (*n* = 105), all VACV-naïve	Netherlands	Days 0, 28, 56	ELISA, PRNT	77% developed detectable MPXV nAbs, but titres were low. IgG to MPXV antigens was lower compared to VACV antigens.
	Grüner et al., J Infect Dis, 2024 [31]	Observational Cohort Study	2× MVA	Adults (*n* = 17), all PWH, 11 VACV-experienced	Germany	Months 0, 3	ELISA, live-virus microneutralisation assay	Enhanced IgG and nAb responses in VACV-experienced.
B Cell	Oom et al., Journal of Virology, 2025 [22]	Observational Cohort Study	2× MVA	Adults (*n* = 159), including PWH, 31% prior VACV vaccination	USA	Weeks 0, 3; months 3, 9	Memory B cell ELISpot	Memory B cells were detectable in low proportions (less than 40%) at 1 year.
	Cohn et al., Lancet Infect Dis., 2023 [32]	Observational Cohort Study	1 or 2× MVA	Adults (*n* = 10)	USA	6–60 days post-vaccine	Transcriptomics (single-cell RNAseq)	MVA induced limited B cell activation with negligible gene-level plasmablast and antibody responses.
T Cell	Collier et al., JAMA, 2024 [17]	Observational Cohort Study	1 or 2× MVA	Adults (*n* = 45)	USA	Weeks 0, 3; months 3, 6, 9, 12	Flow cytometry-based ICS against VACV	Low CD4+ and CD8+ T cell responses to VACV at 9 months.
	Drennan et al., Lancet Microbe, 2025 [19]	Observational Cohort Study	2× MVA	Adults (*n* = 34), HIV-negative	United Kingdom	Days 0, 1, 14, 28 (post-dose 1), 28, 90 (post-dose 2)	ELISpot, AIM assay	Peak CD4+ and CD8+ T cell responses were seen by day 14.
	Matusali et al., Journal of Infection, 2024 [21]	Observational Cohort Study	1 or 2× MVA	Adults (*n* = 50), 42% PWH, 50% VACV-experienced	Italy	Months 0, 1, 6, 12	IFN-γ ELISpot	T cell responses were robust and durable in both groups for up to one year.
	Mazzotta et al., eClinicalMedicine, 2024 [24]	Observational Cohort Study	1× MVA VACV-naïve, 2× MVA VACV-experienced	Adults (*n* = 164), including 46% PWH	Italy	Months 0, 1	IFN-γ ELISpot	2 doses led to stronger T cell responses.
	Crandell et al., Lancet Microbe, 2025 [29]	Observational Cohort Study	2× MVA	Adults (*n* = 111), including VACV-experienced (*n* = 36)	USA, Brazil, Portugal	Up to 11 months	Flow cytometry, OPXV peptide stimulation	T cell cross-reactivity to MPXV was robust and long-lasting but declined with age.
	Cohn et al., Lancet Infect Dis., 2023 [32]	Observational Cohort Study	1 or 2× MVA	Adults (*n* = 10)	USA	Up to 2 months	AIM/ICS T cell assays	MVA induced robust CD4+ and CD8+ T cell responses.
	Grüner et al., J Infect Dis, 2024 [31]	Observational Cohort Study	2× MVA	Adults (*n* = 17), all PWH	Germany	Months 0, 3	AIM assay	CD8+ T cell responses increased after the second MVA dose, but CD4+ responses were modest.
	Sisteré-Oró et al., J Med Virol, 2024 [33]	Observational Cohort Study	1 or 2× MVA	Adults (*n* = 24), all PWH	Spain	Days 0, 28	ELISpot, ICS,	Intradermal vaccination induced stronger T cell responses than subcutaneous. CD4+ count correlated with response.

Abbreviations: ELISA (enzyme-linked immunosorbent assay), PRNT (Plaque Reduction Neutralisation Test), AIM (activation-induced marker), ICS (intracellular cytokine staining), Ab (antibody), nAb (neutralising Ab), ECL (electrochemiluminescence), VACV (vaccinia virus), MPXV (monkeypox virus).

**Table 2:** Orthopoxvirus antigens evaluated in mpox immunogenicity studies.

Virion Form	MPXV Antigen	VACV Homologue (% Homology)	Known/Proposed Function	Assay Type and Study
EEV	B6R	B5R (95.9%)	Envelope glycoprotein required for efficient cell spread, complement control	Anti-MPXV B6R IgG (ELISA) [17,19,29,55] Anti-VACVB5 IgG (ELISA) [29] Anti-MPXV B6R IgG, Anti-VACVB5 IgG (Luminex^®^) [19] Anti-MPXV B6R IgG, Anti-VACVB5 IgG (Electrochemiluminescence Assay) [16]
	A35R	A33R (96.1%)	Envelope glycoprotein, needed for the formation of actin-containing microvilli and cell-to-cell spread	Anti-MPXV A35R IgG (Luminex^®^) [19,22] Anti-VACVA33R IgG (Luminex^®^) [19] MPXV A35R-specific Memory B Cell (ELISpot) [22] Anti-MPXV A35R IgG (ELISA) [17,29,32,55] Anti-VACVB5 A33R IgG (ELISA) [29]
	B2	A56 (93%)	Type I membrane glycoprotein haemagglutinin	Anti-MPXV B2 IgG (Luminex^®^) [19]
IMV	A29L	A27L (93.6%)	Surface membrane fusion protein, binds cell surface heparan	Anti-MPXV A29L IgG (ELISA) [17,29,32,55] Anti MPXVA29L, Anti-VACV A27L IgG (Luminex^®^) [19] Anti-VACV A27L IgG (ELISA) [29]
	H3L	H3 (92.3%)	Heparan-binding surface membrane protein, attaches to the cell surface by binding to glycosaminoglycan (GAG)	Anti-MPXV H3L IgG (Luminex^®^) [19,22] Anti-MPXV H3L IgG (ELISA), MPXV H3L-specific Memory B Cell (ELISpot) [22] Anti-H3L IgG (ELISA) [17,20]
	M1R	L1 (98.8%)	Surface membrane protein, mediates virus entry into cells independently of GAG	Anti-MPXV M1R IgG (ELISA) [17,29,55] Anti-MPXV M1R IgG (Luminex^®^) [19] Anti-VACV L1R IgG (ELISA) [29]
	A27L	Deleted from MVA-BN genome	Surface membrane protein	Anti-MPXV A27L IgG (Electrochemiluminescence Assay) [16] Anti-MPXV A27L IgG (Luminex^®^) [19] Anti-MPXV A27L IgG (ELISA) [32]
	E8L	D8 (94.7%)	Surface membrane protein, binds cell surface chondroitin sulfate, IMV adsorption to cell surface	Anti-MPXV E8L IgG (Luminex^®^) [19] Anti-MPXV E8L IgG (ELISA) [29,32] Anti-VACV D8 IgG (ELISA) [29]
	A5	A4 (95%)	Immunodominant virion core protein	Anti-MPXV A5 IgG (Luminex^®^) [19]

Abbreviations: IMVs, intracellular mature virions; EEVs, extracellular enveloped virions; MPXV, monkeypox virus; VACV, vaccinia virus. MPXV antigens based on clade I MPXV reference genome Zaire-96-I-169 (NCBI:txid619591). VACV antigens based on the VACV Copenhagen strain genome (NCBI:txid10249). Homology between MPXV and VACV antigens was calculated using the NCBI BLASTp tool [7]. The known function of MPXV and VACV antigens is summarised [49,53,56,57].
